# Duality picture of Superconductor-insulator transitions on Superconducting nanowire

**DOI:** 10.1038/srep27001

**Published:** 2016-06-17

**Authors:** Kazumasa Makise, Hirotaka Terai, Yukihiro Tominari, Shukichi Tanaka, Bunju Shinozaki

**Affiliations:** 1 National Institute of Information and Communications Technology, Kobe 651-2492, Japan; 2 Department of Physics, Kyushu University, Fukuoka 819-0395, Japan

## Abstract

In this study, we investigated the electrical transport properties of niobium titanium nitride (NbTiN) nanowire with four-terminal geometries to clarify the superconducting phase slip phenomena and superconducting-insulator transitions (SIT) for one-dimensional superconductors. We fabricated various nanowires with different widths and lengths from epitaxial NbTiN films using the electron beam lithography method. The temperature dependence of resistance *R*(*T*) below the superconducting transition temperature *T*_c_ was analyzed using thermal activation phase slip (TAPS) and quantum phase slip (QPS) theories. Although the accuracy of experimental data at low temperatures can deviate when using the TAPS model, the QPS model thoroughly represents the *R*(*T*) characteristic with resistive tail at low temperatures. From the analyses of data on *T*_c_, we found that NbTiN nanowires exhibit SIT because of the change in the ratio of kinetic inductance energy and QPS amplitude energy with respect to the flux-charge duality theory.

The state-of-the-art superconducting quantum computer consists of superconductor-insulator-superconductor tunnel junctions called Josephson junctions because it is a commonly used for superconducting digital circuits and quantum qubit for quantum computing devices. Recently, one-dimensional (1D) superconducting nanowires (SNWs) are being considered to develop superconducting computing devices^1^,^2^. In order to realize novel devices using nanowires, it is necessary to clarify the superconducting transport characteristics that depend on the disorder, wire length *L*, width *w,* and many other parameters. In such low-dimensional superconductors, quantum effects slightly influence the superconducting characteristics. For instance, on when the film thickness *d* of the two-dimensional (2D) specimens decreases, the superconducting transition temperature *T*_c_ is gradually depressed and then the superconductivity disappears for films thinner than the critical thickness *d*_c_, where the 2D superconductor-Insulator transitions (SIT) occurs^3^,^4^,^5^,^6^. *T*_c_ depression is expected by enhanced Coulomb repulsion due to increase of disorder^7^. As for other tuning parameters of SIT, we can consider not only the film thickness but also the external magnetic field and carrier density^8^. The SIT originates from the fluctuations of an amplitude and/or a phase of superconducting order parameter. Especially, SNWs can be strongly affected by thermally activated phase slip (TAPS)^9^,^10^ and/or quantum phase slips(QPS)^11^,^12^ which play an important role in the properties of SNWs. The effect of TAPS to resistance *R*(Ω) sharply decays as a result of the temperature drop below *T*_c_. On the other hand, the QPS represents the residual resistance required to suppress the superconductivity at *T* = 0 K. Numerous studies have been conducted on the various materials that can be used for SNWs^13^,^14^,^15^,^16^,^17^,^18^. However, there still are fundamental problems in determining the effect of TAPS and QPS on the temperature dependency of resistance *R*(*T*) because SNW specimens with the same material can present different *R*(*T*) characteristics. Further, some investigations show no evidence of QPS behavior at very low temperatures^14^,^17^,^18^.

Another attractive subject of investigation for SNWs is clarifying some critical values corresponding to SIT, namely, the normal state critical resistance *R*^N^_c_, (*R*^N^/*L*)_c_, or other characteristics. Several research groups have reported the characteristics of *R*(*T*) below *T*_c_ and SIT for MoGe SNWs^13^,^17^,^18^. For (*R*^N^/*L*)_c_, separating 1D specimens into superconducting and insulator phases, the (*R*^N^/*L*)_c_ takes the almost same values in the region 30 < (*R*^N^/*L*)_c_ < 100. On the other hand, *R*(*T*) characteristics show different behaviors owing to the TAPS or QPS mechanisms. Although Marković *et al.*^19^ and Tinkham *et al.*^13^ observed broad transition with a resistive tail due to the QPS at low temperatures, others^14^,^17^ fit the TAPS model to data without the resistive tail. As for *R*^N^_c_, it is expected to take the superconducting quantum resistance *R*_Q_ = *h*/4e^2^ ≈ 6.45 KΩ for Cooper pairs^20^,^21^,^22^. This suggestion is conformed for short wires by phenomenological model^23^ and experimentally confirmed for specimens with a length of *L* < 200 nm^14^. However, the data for longer specimens are inconsistent in that they exhibit *R*^N^_c_ > *R*_*Q*_ and show superconductivity^15^,^24^. Thus, there is no consensus between experiments and theories on the exact role of SIT of 1D-SNWs.

Recently, Mooij *et al.* proposed an idea that the concept of flux-charge duality can relate the QPS with Josephson tunneling if the roles of phase and charge are interchanged^1^. They discussed the crossover value between superconducting and insulating states at low temperatures as a function of ratio *α* = *E*_s_/*E*_L_, where *E*_s_ and *E*_L_ are the QPS energy and inductive energy, respectively. Increasing *E*_s_ leads to a transition from inductively superconducting regime where *E*_L_ ≫ *E*_s_ to a capacitive insulating regime where *E*_s_ ≫ *E*_L_. Further, they succeeded in showing the phase boundary between the superconducting and insulating state of data for MoGe SNWs^14^ by assuming *α* = 0.3.

To observe the quantum phase slip in SNWs, specimens are required to be homogeneous and satisfy the condition *d*,*w* ≤ *ξ*, where *ξ* is superconducting coherence length. Further, poor links due to inhomogeneities in the superconducting wires can cause residual resistance at low temperatures as pointed by Altomare *et al.*^15^ In the present report on homogenous nanowires, the *R*(*T*) characteristics of NbTiN SNWs in a broad range of the *R*^N^/*L* were investigated from the viewpoint of QPS mechanisms. We analyzed the data from the superconducting and insulating phase diagrams based on the flux-charge duality model using the relation (*R*^N^/*R*_Q_)/(*L*/*ξ*) versus *L*/*ξ* with a suitable parameter *α* and other parameters in theories^1^.

## Experimental Procedure

Superconducting NbTiN films were firstly prepared by deposition at ambient temperatures on (100)-MgO substrates by DC reactive magnetron sputtering. The background pressure of the chamber was maintained below 2.0 × 10^−5^ Pa. The relative amounts of argon and nitrogen were controlled by mass flow controller during sputtering. The total pressure was maintained at 2 mm torr and the substrate was not heated intentionally during deposition. Details of preparation procedures and films quality of NbTiN thin films are previously reported^25^. The NbTiN SNWs were fabricated from 2D films with *d* = 5 nm by a conventional e-beam lithography method and a reactive ion etching method with CF_4_ plasma. The ranges of *L* and *w* of nanowires are 250 ≤ *L* ≤ 1000 nm and 10 ≤ *w* ≤ 30 nm, respectively. To eliminate the influence of the contact resistance, measurements of transport properties were performed by four-probe method. The normal state resistance, *R*^N^, is defined as the sample resistance at 20 K. The *T*_C_ and *H*_C2_ were defined as the point at which the *R*^N^ reached half its value.

## Results and Discussion

**Figure 1:**
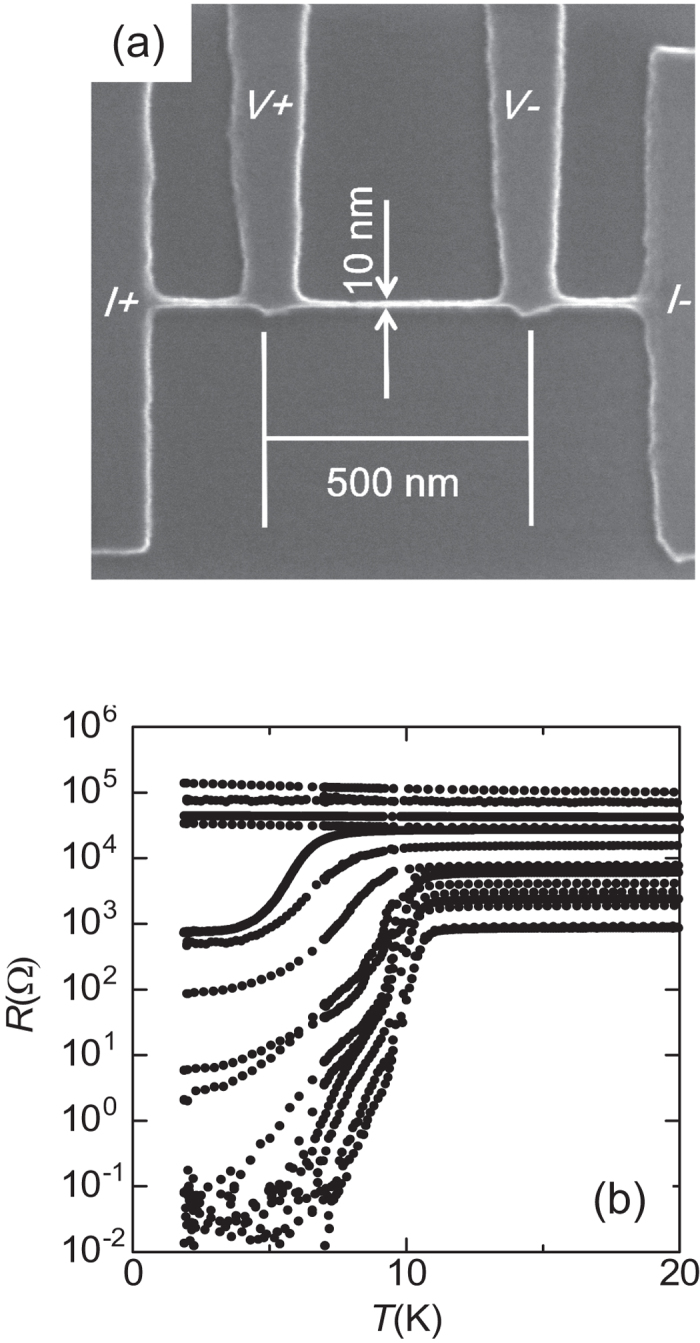
(**a**) Scanning electron microscopy image of a NbTiN SNW with 10 nm-width. (**b**) *R*(*T*) for in series of NbTiN SNWs within ranges of 250–1000 nm length *L* and 10–30 nm width *w*.

Figure 1(a) shows the scanning electron microscopy image of typical NbTiN SNWs. Figure 1(b) presents the characteristics of *R*(*T*) for various SNWs with different values of *w* and *L*. Superconducting SNWs that have *dR*/*dT* > 0 under low temperatures and low *R(Ω)* characteristics, experience the initial drop of *R(Ω)* almost at the same temperature owing to the superconducting transition. An increase in *R*^N^ causes *T*_c_ and the residual resistance to monotonically decrease and increase, respectively. Prior to the detailed discussions on the SIT of 1D specimen from a viewpoint of quantum phase transition, we will present some transport properties of the present SNWs from the characteristics of low dimensional superconductors.

**Figure 2:**
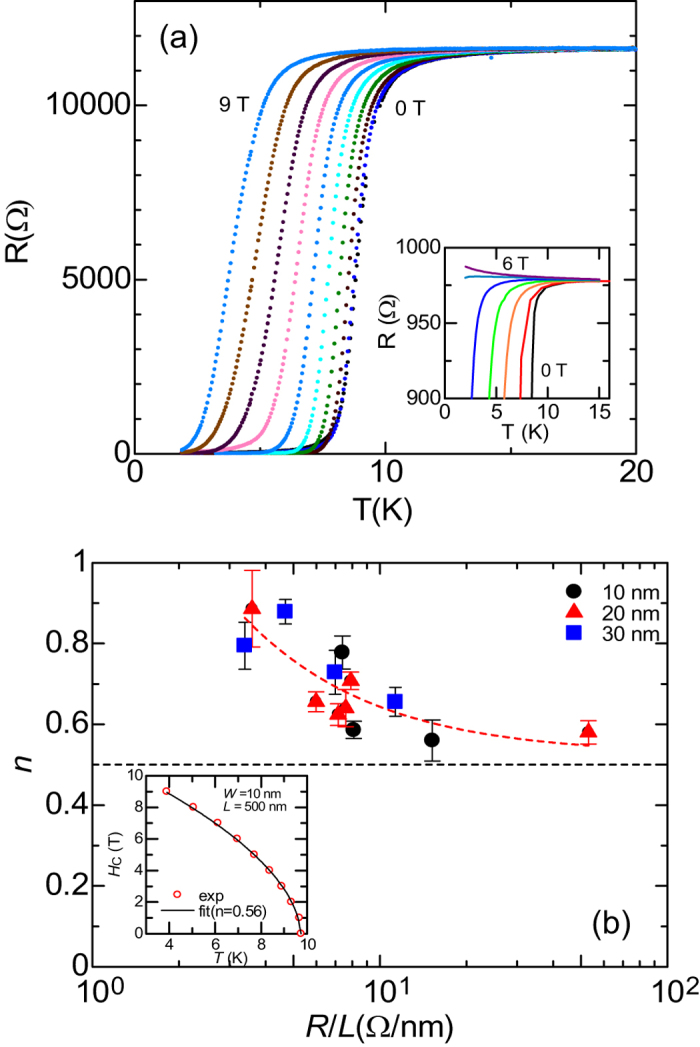
(**a**) *R*(*T*) for the NbTiN SNW with *d* = 5 nm, *w* = 20 nm and *L* = 500 nm under the magnetic field ranging from 0 to 9 Tesla with a division of 1 Tesla. Inset shows the *R*(*T*) for the NbTiN film with the same thickness of the nanowire under the magnetic field. (**b**) *R*^N^/*L* dependence of the index *n* in Eq. (1). Inset shows the typical *H*_c2_(*T*) for the nanowire with *w* = 10 nm. Where the solid line is represented by calculating Eq. (1).

Figure 2(a) shows a typical *R*(*T*) of the NbTiN SNWs with *L* = 500 nm and *w* = 20 nm at various external magnetic fields. With an increase in *H*, *T*_c_ monotonically decreases without field-tuned SIT even at *H* = 9 T. On the other hand, as shown in the inset, the 2D NbTiN with almost the same thickness (≈5 nm) shows that the field-tuned SIT occurs around 5–6 T where many vortexes in the film appear to transition into super-fluid states of vortexes in the dirty boson scenario^26^,^27^,^28^. The present result for NbTiN SNWs suggests that this nucleation of the vortex in the superconducting state is inhabited because of the 1D-restricted geometry of the nanowire. We consider the dimensionality and size effects of the nanowire on the upper critical magnetic field *H*_c2_. The suppression of superconductivity by perturbations is given by the relation, 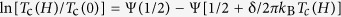, where Ψ(*x*) is the digamma function and δ is the pair-breaking strength which depends on the dimensionality of the specimen and the direction of the external magnetic field^29^. Expanding the function 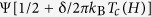 around *x* = 1/2, we obtained the relation *k*_B_[*T*_c_(0) − *T*_c_(*H*)] = δ*π*/4in the temperature range near *T*_c_(0), where δ is given by δ = *DeH*/*c* and δ = *DeH*^2^*d*^2^/6*ħc*^2^ for fields perpendicular and parallel to the surface of 2D specimen with *d* < *ξ*(0), respectively, and *D* is the diffusion constant. From the above relation, *H*_c2_(*T*) near *T*_c_(0) ≡ *T*_c0_ is given by


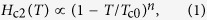


where the index *n* is 1 and 1/2 for magnetic fields perpendicular and parallel to the surface, respectively. When the expression for the parallel case is approximately applied to SNWs, it is expected that *n* approaches 1/2 with *w* ≈ *ξ*(0). By using the Eq. (1), we obtained the index *n* for each SNW. Figure 2(b) shows the *R*/*L* dependence of *n* for NbTiN SNWs, where the dotted line is the reference point. It can be seen that the index *n* approaches 0.5 with an increase in the *R*/*L* ratio. The inset shows the typical data of *H*_c2_(*T*) for the SNW with *w* = 10 nm and *L* = 500 nm. The solid line shows Eq. (1) with *n* = 0.56. These results indicate the 1D transport property of the present SNWs.

To clarify the mechanism of the resistive tail for NbTiN SNWs at low temperatures shown in Fig. 1(b), we analyzed the *R*(*T*) and the voltage-current characteristics in a broad temperature range. The fluctuation of the superconducting order parameter *ψ*(*r*) plays an important role in the transport properties of the 1D superconductor. The magnitude of *ψ*(*r*) vanishes at some points in the SNWs owing to the fluctuation, and it recovers the phase slip by 2π. There are two mechanisms for the phase slip, TAPS and QPS. According to the TAPS model, *dV*/*dI* and *R*_TAPS_(*T*) are expressed by^9^,^10^


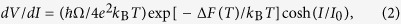


and


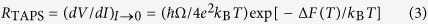


where Ω = (*L*/*ξ*)(Δ*F*/*k*_B_*T*)^1/2^(1/*τ*_GL_), Δ*F*(*T*) = 0.83*k*_B_*T*_c_(*R*_Q_/*R*^N^)(*L*/*ξ*(0)(1 − *T*/*T*_c_)^3/2^ is the energy barrier, *I*_0,TAPS_ = (4*ek*_B_/*h*)*T* and *τ*_GL_ = [*πħ*/8*k*_B_(*T*_c_ − *T*)] is the relaxation time of G-L equation. On the other hand, *R*_QPS_ (*T*) is expressed as


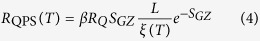


where *S*_*GZ*_ = 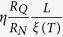 is the normalized unit measured by *R*_Q_ and *ξ*(*T*), and *β* and *η* are fitting parameters held constant on the order of unity^30^,^31^. For *ξ*(*T*), the expression 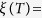

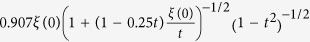
 is adopted^32^. The *dV*/*dI* is also given by


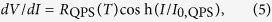


where *I*_0,QPS_ is expected to have a different temperature dependence from *I*_0,TAPS_ = (4*ek*_B_/*h*)*T*.

**Figure 3:**
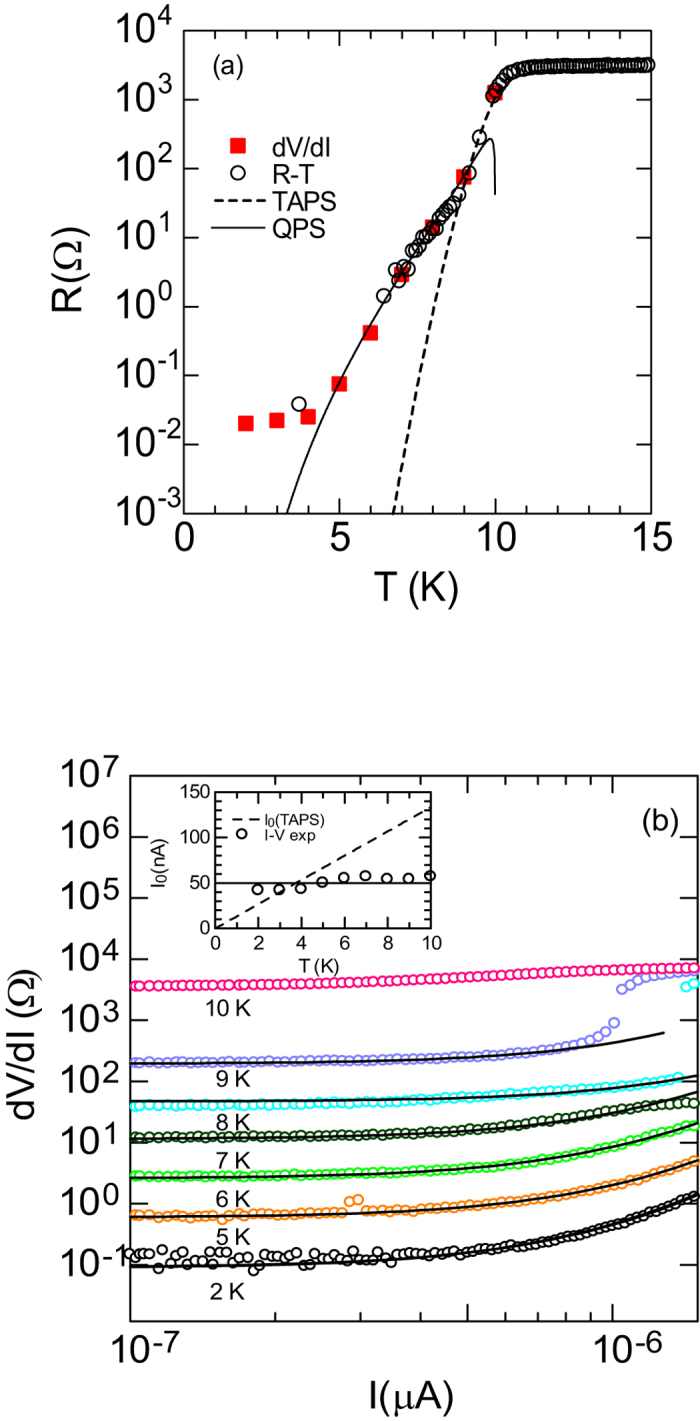
(**a**) *R*(*T*) for the NbTiN SNW with *d* = 5 nm, *w* = 10 nm and *L* = 500 nm. Open circles are resistances from the low bias current measurement and squares show resistances obtained by applying the theory to the data of *dV*/*dI* in (**b**). Dotted and solid lines are calculated using Eq. (2) and Eq. (4), respectively. (**b**) Bias current dependence of differential resistance under various temperatures below *T*_c_. Solid lines are calculated using Eq. (3). The inset shows the temperature dependency of *I*_0,QPS_. The dashed line shows *I*_0_(*T*) = (4*ek*/*h*)*T* predicted by the TAPS theory. Solid line *I*_0_ = constant is a reference point.

Figure 3 shows the superconducting transport properties for the NbTiN SNW with *d* = 5 nm, *w* = 10 nm, *L* = 500 nm, *T*_c_ = 10.0 K, and *R*^N^ = 5.0 kΩ. Figure 3(a) shows *R*(*T*) from the measurements of current-bias (○), and the d*V*/d*I* (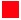) at *I* ≈ 0 shown in Fig. 3(b). The calculation made using the TAPS model shown by the dashed line (---) cannot explain *R*(*T*) characteristic except for temperatures close *T*_c_ and unfortunately, the theory strongly deviates from the data below 8 K. This discrepancy suggests that transport properties of the present NbTiN SNWs are incompatible with TAPS theory at low temperatures. The solid line is calculated using Eq. (4) with parameters of *ξ*(0) = 8 nm, *β* = 0.0013, and *η* = 0.024 in order to fit theory with *R*(*T*) data under a broad temperature range. The calculation using the QPS model agrees accurately with the resistive tail in the range of 5 magnitude orders. Figure 3(b) represents the *I–V* characteristics at temperatures of 2 K, 5 K, 6 K, 7 K, 8 K, and 9 K. The *I–V* curves have shown nonlinear characteristic in the superconducting region below the 9 K. This *I* dependence of *dV*/*dI* at each temperature agrees well with the term of cosh(*I*/*I*_0,QPS_) in the Eq. (5) as shown by the solid lines^33^. From the fitting procedure, we obtained the temperature dependences of *R* and *I*_0,QPS_. The inset in Fig. 3(b) shows the *I*_0,QPS_(*T*), which is almost independent of temperature as shown by the dotted line. This discrepancy with the TAPS model shown by the solid line is consistent with the experimental result that *R*(*T*) cannot be explained by the TAPS model.

**Figure 4:**
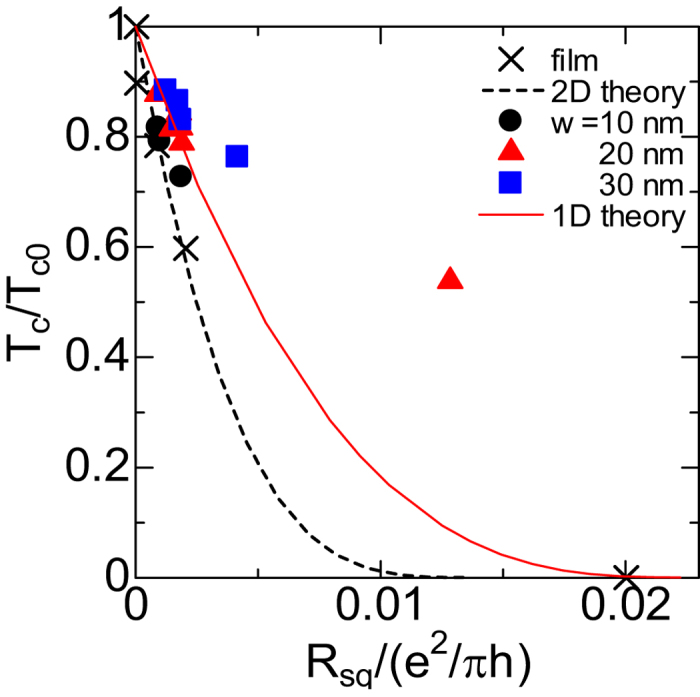
*R*_sq_ dependence of *T*_c_ for NbTiN film and nanowire specimens. Dotted and solid lines are calculated from theories based on the dynamically enhanced Coulomb repulsion in dirty systems for the film and nanowire, respectively.

**Figure 5:**
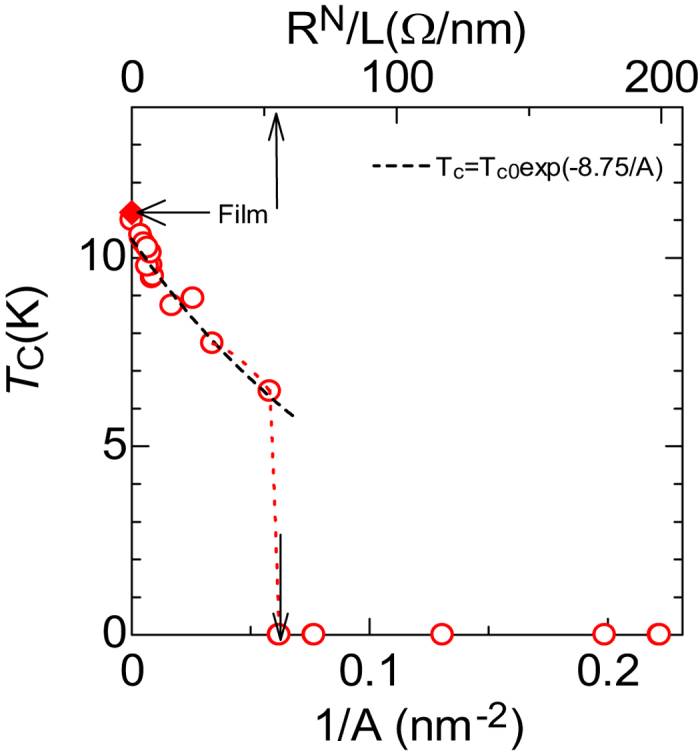
*R*^N^/*L* (upper horizontal axis) and 1/*A* (down horizontal axis) dependencies of *T*_*c*_ for NbTiN SNWs. Mark (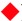) represents the *T*_c_ for 2D specimens. The broken line represents *T*_c_ = *T*_c0_exp(−8.75/*A*) determined from data. The dotted line is the reference point. Arrows, ↑ and ↓ indicate the SIT points.

Before investigating the *R*^N^/*L* ratio dependence on *T*_c_ in order to clarify the SIT of NbTiN SNWs, we will analyze the data using the theory based on the dynamically enhanced Coulomb repulsion competing the attractive interactions between electrons^7^. The theoretical expression for *T*_c_ is given by a simple formula as a function of sheet resistance *R*_sq_ (resistance for unit area) with the parameter 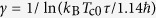, where *τ* is the electron elastic scattering time. Figure 4 represents the *R*_sq_ dependence of *T*_c_ for both nanowire specimens and 2D specimens. *T*_co_ values are expected to be independent of *w* and *L* as shown in Fig. 5, because the *T*_c_ on the vertical axis is normalized by *T*_c0_ = 11.0 K of pure 2D films. Although *R*_sq_ of 2D specimens was controlled by changing the thickness, *R*_sq_ of SNW specimens with different *w* was controlled by changing the length *L* and by keeping the thickness constant at ≈5 *nm* for all SNWs. The dotted line is calculated by using the theory for impure 2D system^5^ in order to fit the data (×) with a parameter 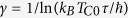. The good agreement between the theory and data suggests that the *T*_c_ depression of 2D NbTiN films is determined by the decrease in the amplitude of the superconducting order parameter that belongs to the system confirming the fermionic scenario. As for 2D NbN and NbTiN films, we have already investigated transport properties on the fluctuations and SIT^5^,^28^. We reported that the critical sheet resistance *R*_c_ is approximately 2.2 kΩ and superconducting suppression mechanism can be explained by the localization theory. On the other hand, data for SNW specimens in the range of 10 nm ≤ *w* ≤ 30 nm do not collapse on the unique line calculated by the theory^7^. In addition, the depression of *T*_c_ cannot be explained only by enhanced Coulomb interaction in impure superconductors.

Figure 5 shows *T*_c_ as functions of [*R*(20 K > *T*_c_) ≡ *R*^N^]/*L* (upper axis) and 1/*A* (lower axis), where the cross section area *A* is derived from the relation *R*^N^/*L* = *ρ*/*A* using the resistivity value of *ρ* = 0.9 μΩm determined before the fabrication of 2D films. The *T*_c_ was defined as the temperature at which the resistance takes one half of *R*(20 K). For specimens with different size of *w* and *L*, data of *T*_c_ concurs well onto a single curve in a broad *R*^N^/*L* (or 1/*A*) range. This fact expresses that the quantity *R*^N^/*L* ratio is a sufficient parameter to describe the impurity dependence of *T*_c_ for the 1D system and for 2D system in the case *R*^N^ is replaced with *R*_sq._ The *T*_c_ slowly decreases below the value *R*^N^/*L* ≈ 50Ω/nm when *R*/*L* increases, and rapidly decreases in the range above 50–60 Ω/nm. For a relation between *T*_c_ and *R*^N^/*L* in Fig. 5, Marković *et al.*^19^ proposed a simple criterion for the crossover value of (*R*^N^/*L*)_c.o_ separating an insulating state from superconducting state. If the wire resistance at *T* = 0 due to the quantum phase slip is comparable to *R*^N^, the resistance drop does not appear to sustain the value of *R*^N^ even at very low temperatures. Using Eq.(4) at *T* = 0, they obtained the normalized resistance due to quantum phase slips^24^ as


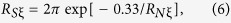


where the resistances *R*_Sξ_ and conductance *R*_Nξ_ are measured in units of quantum resistance *R*_Q_ and length in units of coherence length *ξ*, namely, *R*_Sξ_ = (*R*_S_/*R*_Q_)/(*ξ*/*L*) and *R*_Nξ_ = (*R*^N^/*R*_Q_)/(*ξ*/*L*). From Eq. (6), by equating *R*_Sξ_ to the *R*^N^, we obtain the value (*R*^N^/*L*)_c.o_ = *R*_Q_/13.3*ξ*(0) ≈ 485Ω/*ξ*(0). As for the present series of which *ξ*(0) ≈ 9 *nm*, we obtain (*R*^N^/*L*)_c.o_ ≈ 54Ω/nm. This value is almost the same one denoted by the arrow ↑ separating the superconductor and insulator phases in Fig. 5.

From 1/*A* dependence of *T*_c_ in Fig. 5, we can quantitatively discuss on the critical diameter *D*_c_ of NbTiN SNW at SIT. The dotted line in Fig. 4 represents the relation 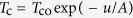, where *T*_c0_ = 11.0 K and *u* = 8.75 *nm*^2^ are the transition temperature of 2D film and the parameter determined from the fitting procedure, respectively. 1/*A* dependency of *T*_c_ has also been reported in Mo_78_Ge_22_ and Mo_50_Ge_50_ SNWs^18^ that is having greater *α* than that of the present NbTiN. If this relation for NbTiN SNW is valid for board range 1/*A*, the *D*_c_ ∝ *u*^1/2^ value is expected to be smaller than ≈2 nm. On the other hand, the *T*_c_(1/*A*) characteristic drastically decreases around 1/*A* ≈ 0.06 *nm*^−2^, giving *D*_c_ ≈ (4*A*/*π*)^1/2^ ≈ 4.6 *nm* denoted by the arrow ↓, which is approximately half of the 2D NbTiN coherence length *ξ*(0) ≈ 9 *nm* determined from the relation 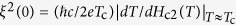
6. This estimation suggests that the restricted geometry of SNW allows smaller critical diameter than *ξ*(0) for 2D specimens.

**Figure 6:**
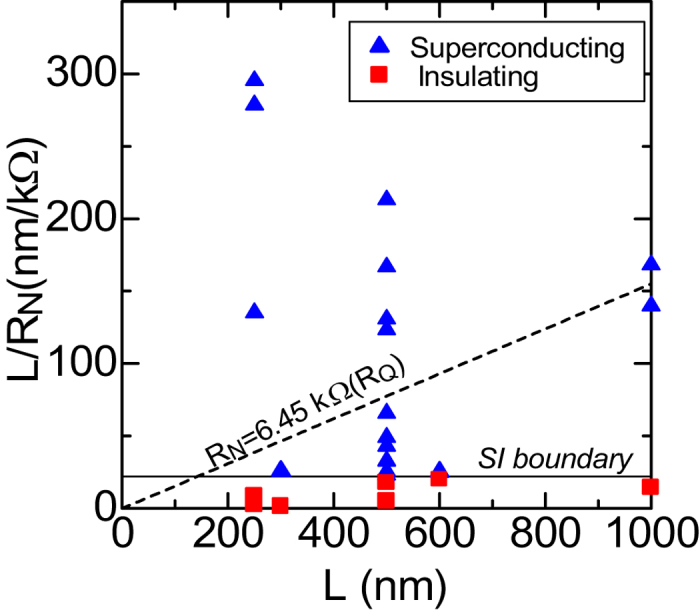
Phase diagram of *R*^N^/*L* versus *L* for NbTiN SNWs. Squares (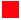) and triangles (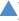) represent insulating and superconducting wires, respectively. The solid line indicates the boundary of SIT estimated from experimental results of *R-T* characteristics. The dotted line is the boundary of SIT expected from the Chakravarty-Schmid-Bulgadaev theory. (see text.).

Now, we will discuss of size dependence of *T*_c_ and SI phase diagram for the present SNWs. To clarify the 1D SIT mechanisms of NbTiN SNWs, we illustrated the SI phase diagram in Fig. 6 according to the Chakravarty–Schmid–Bulgadaev theory based on the interaction of QPS and dissipative environment^20^,^21^,^22^. Such a relation between *L* and *L* /*R*^N^ has been reported for MoGe SNWs^14^. The author claimed that the SIT boundary is given by a condition *R*^N^ = *R*_Q_ = 6.45 kΩ. However, the present NbTiN SNWs specimens with *L* > 500 *nm* do not satisfy this condition as shown by the dashed line, that is, specimens with *L* > 500 *nm* show superconductivity though *R*^N^ is larger than *R*_Q_. Although the *R*(*T*) characteristic of NbTiN SNWs can be explained by the QPS theory as discussed in Figs 4 and 5, the phase diagram shown in Fig. 6 suggests that the SI boundary depends on the length of the nanowire.

QPS and the Josephson effects in SNWs are related to each other by a concept of duality transformation. According to this concept^1^, the SIT is determined by the ratio between the strength of QPS amplitude energy *E*_S_ and SNW inductive energy *E*_Li_. Both energies are given by *E*_*S*_ = *eV*_0_/*π* = *a*(*L*/*ξ*)*k*_B_*T*_c_(*R*_*Q*_/*R*_*ξ*_)exp(−*bR*_*Q*_/*R*_*ξ*_) and*E*_*Li*_ = *ϕ*_0_/2*L*_*i*_ = 17.4*k*_*B*_*T*_*c*_(*R*_*Q*_/*R*^*N*^), where the *R*_*ξ*_ = *R*^N^*ξ*/*L* is the resistance of the SNW over an appropriate length, *L*_*i*_ = 0.18*ħR*^N^/*k*_B_*T*_c_ is the kinetic inductance of the wire, Φ_0_ = *h*/2*e* is the flux quantum, and *a* and *b* are constants of order one. According to Mooij *et al.*^1^, it is expected that SIT occurs at condition *E*_*s*_/*E*_*Li*_ = (*aλ*^2^/17.4)exp(−*b*/*r*_*ξ*_) = *α*_c_, where *λ* = *L*/*ξ*_*i*_ and *r*_*ξ*_ = *R*_*ξ*_/*R*_Q_(=*R*^N^*ξ*/*R*_Q_*L*) are the normalized length and resistance, respectively. From the equation for *E*_*s*_/*E*_*Li*_, we obtain the *λ* dependence of *r*_*ξ*_ as


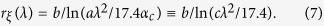


**Figure 7:**
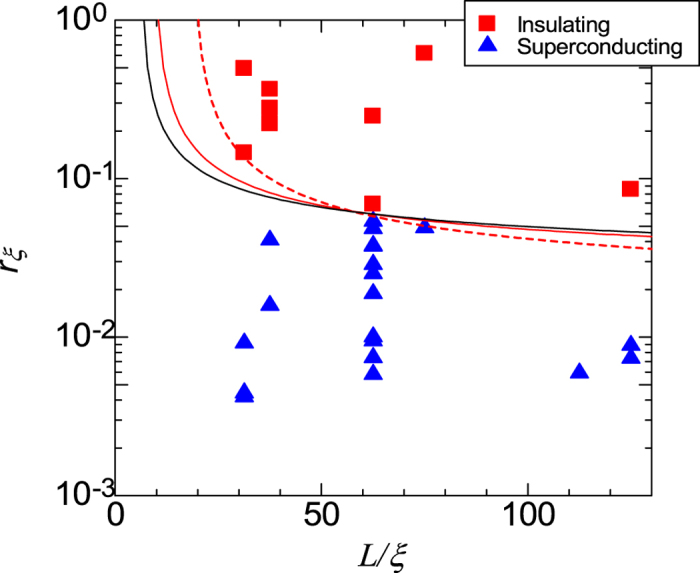
Phase diagram of *L/ξ*(=*λ*) versus *r*_*ξ*_ = (*R*^*N*^/*R*_*Q*_)/(*L/ξ*) for NbTiN SNWs. (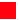) and (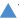) are the same marks in Fig. 6. Lines denoted by (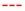), (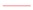) and (^____^) are calculated from Eq. (7) with input parameters (*b*, *c*) = (0.14, 0.05), (*b*, *c*) = (0.23, 0.2) and (*b*, *c*) = (0.28, 0.5), respectively.

Figure 7 shows the *r*_*ξ*_(*λ*) for all same NbTiN SNWs shown in Fig. 6, where ξ = 8 nm is used. To show a boundary separating the superconducting phase from the insulator phases, we calculate *r*_*ξ*_(*λ*) from Eq. (7) with input parameters *b* and *c*. Although data are not so large to make clear the boundary, we attempt to find reasonable values for *b* and *c* assuming that the theoretical line must go through a reliable point of *r*_*ξ*_(*λ*) ≈ 0.6 at *λ*(=*L*/*ξ*) ≈ 62 for analysis. The red-broken, red-solid, and black-solid lines are typically calculated from Eq. (7) to divide the data into superconducting and insulator phases with the use of parameters (*b*, *c*) = (0.14, 0.05), (*b*, *c*) = (0.23, 0.2) and (*b*, *c*) = (0.28, 0.5), respectively. When we take into account the theoretical suggestions that the strength *p* of three parameters *a*, *b* and *α*_c_ is given as 0.1 < *p* < 1. Further, the first combination of (*b*, *c*) = (0.14, 0.05) corresponds to (*α*_c_, *a*, *b*) = (0.025, 0.5, 0.14) due to the definition *c* = *a*/*α*_c_ in Eq. (4). This critical ratio *α*_c_ = 0.025 is very small compared with the theoretical suggestion. On the other hand, other combinations shown by solid and dotted lines give reasonable values for *a* and *b*, namely, 0.1 < *a* < 0.25 and 0.23 < *b* < 0.28 for *α*_c_ = 0.5. These values are comparable to those analyzed by Mooij *et al.* for Mo/Ge SNWs data.

## Conclusion

We investigated the transport properties of superconducting NbTiN SNWs in a wide range of *R*^N^/*L* using four-probe method to eliminate the contact resistance. The *R*(*T*) characteristic with resistive tail below *T*_c_ for SNWs with high values of *R*^N^/*L* can be well explained by the QPS theory. With the increasing *R*^N^/*L*, the behavior of the *R*(*T*) characteristic changes from superconducting to insulating. *R*(*T*) exhibits superconducting-insulator transition near (*R*^N^/*L*)_c.o_ ≈ 60 Ω/nm, which agrees well with the prediction based on the QPS model by Marković *et al.* As for the S-I phase boundary for the NbTiN SNWs, the phase diagram *L*/*R*^N^ vs. *L* is inconsistent with Chakravarty–Schmid–Bulgadaev theory, which has succeeded in describing the SIT for short MoGe SNWs. On the other hand, the analysis based on the model for the SNW which is being dual element upto Josephson junction, suggests that the separation of the superconducting and insulator phases may be controlled by the ratio of QPS amplitude energy *E*_S_ and inductive energy of SNW *E*_Li_, *E*_s_/*E*_Li_. For the present NbTiN series, we observed that SIT may occur at 0.2 < *E*_s_/*E*_Li_ < 0.5.

## Additional Information

**How to cite this article**: Makise, K. *et al.* Duality picture of Superconductor-insulator transitions on Superconducting nanowire. *Sci. Rep.* **6**, 27001; doi: 10.1038/srep27001 (2016).
